# Acoustic Traits of Three Hawaiian Honeycreepers in a Fragmented Landscape

**DOI:** 10.1002/ece3.71919

**Published:** 2025-08-11

**Authors:** Esther Sebastián‐González, Jomar M. Barbosa, Pablo Montoya‐Bernabeu, Patrick J. Hart

**Affiliations:** ^1^ Department of Ecology University of Alicante Alicante Spain; ^2^ Instituto Multidisciplinar para el Estudio del Medio “Ramón Margalef” University of Alicante Alicante Spain; ^3^ Department of Applied Biology University Miguel Hernández Elche Spain; ^4^ Department of Biology University of Hawaiʻi at Hilo Hilo USA

**Keywords:** ‘Apapane, ‘I‘iwi, Circuitscape, connectivity, Hawai‘i ‘Amakihi, song, vocalization

## Abstract

Habitat fragmentation is a global issue impacting animal populations and is primarily caused by human activities. The main consequences of habitat fragmentation are habitat loss and patchiness, although fragmentation can also increase habitat diversity and landscape complementation. While the impact of habitat fragmentation on animal communities is heterogeneous due to positive and negative effects on biodiversity, its influence on the diversity of learned cultural traits within those communities remains less understood. This study assesses the acoustic traits of three species of native Hawaiian honeycreepers (‘Apapane *Himatione sanguinea* , ‘I‘iwi *Drepanis coccinea* and Hawaiʻi ‘Amakihi *Chlorodrepanis virens* ) across a naturally fragmented landscape. Using automatic recording units, we gathered bird vocalization data from five bird populations across the Big Island of Hawai‘i and used these data to manually identify the distinct syllables vocalized by individuals of the three species. Additionally, we assessed the habitat connectivity between these five populations using a model that considered the land‐cover type, the species' likelihood of occurrence in each land‐cover, the cost of multiple dispersal pathways, and bird susceptibility to a disease constrained by topography. Finally, we compared the acoustic repertoires among the different populations and examined the relationship between their acoustic dissimilarity, geographic distance, and inter‐population connectivity. The results show that the three honeycreeper species have large repertoires (i.e., with 52–215 different syllables), with low overlap among study sites. Acoustic dissimilarity was not related to geographic distance for any species, although inter‐population connectivity determined the acoustic traits of one of the species (‘I‘iwi *Drepanis coccinea*). The study suggests that landscape connectivity plays a variable role in shaping acoustic differences. Overall, this study contributes to a better understanding of the impact of habitat fragmentation on the acoustic diversity of animal communities and highlights the importance of considering cultural traits in conservation efforts.

## Graphical abstract

In this study, we compare bird acoustic diversity in five different populations of Hawaiian honeycreepers using a connectivity model that considered the land use in the space that separated the populations, the species' likelihood of occurrence in each habitat, the cost of multiple dispersal pathways, and bird susceptibility to avian malaria. Interestingly, acoustic dissimilarity was not related to geographic distance, but it significantly correlated with the degree of connectivity for one of the species. The study suggests that acoustic differences for these species occur at small spatial scales and that connectivity among populations, rather than just distance‐based factors, plays a crucial role in shaping acoustic differences.

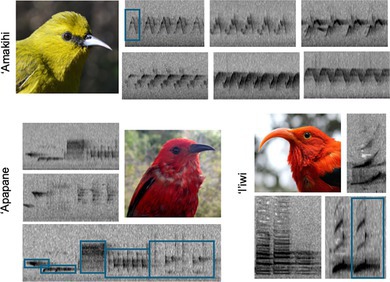

## 1 Introduction

Habitat fragmentation and loss are increasingly significant factors impacting animal populations worldwide (Prugh et al. 2008; Crooks et al. 2017). While natural causes, such as volcanic eruptions, can contribute to habitat fragmentation and loss, human activities are the primary global driver of these impacts (Laurance 2014; Liu et al. 2019). The negative effects of fragmentation on animal communities primarily manifest through two processes: habitat size reduction and isolation (MacArthur and Wilson 1967). Smaller patches have limited capacity to support populations, leading to increased vulnerability to stochastic processes and reduced viability. Additionally, isolated patches pose challenges for species with limited dispersal abilities, as they face difficulties in accessing these patches. Consequently, smaller and more isolated habitats are associated with a diminished number of species (e.g., Bełcik et al. 2020), as well as reduced functional (e.g., Ding et al. 2013) and genetic diversities (e.g., Schlaepfer et al. 2018).

The impact of habitat fragmentation on ecosystems has been extensively documented (e.g., Fahrig 2003; Haddad et al. 2015). However, more recent studies and reviews have challenged the general perception that habitat fragmentation always presents negative impacts on species richness (e.g., Valente et al. 2023; Galán‐Acedo et al. 2024). Therefore, a wide range of responses of the biota to changes in landscape structure and composition is expected. For example, in a review, Fahrig (2017) found 76% of significant positive responses of fragmentation on biodiversity because habitat fragmentation per se can also benefit functional connectivity and habitat diversity, create positive edge effects, stability of disease affections, and reduce species competition. However, the influence of habitat fragmentation on the diversity of cultural learned traits (i.e., behaviors or knowledge that are transmitted from one individual to another through social learning) within these communities is not well understood. One cultural trait that is affected by fragmentation is the acoustic diversity of bird vocalizations (e.g., Pérez‐Granados et al. 2016; Hart et al. 2018), particularly in species known for their ability to learn songs (such as parrots, hummingbirds, and oscine passerines, Petkov and Jarvis 2012). Similar to what happens with genetic diversity, acoustic diversity can be influenced by dispersal rates and cultural drift, resulting in the random loss of song elements and increasing acoustic differences with geographic distance (Potvin and Clegg 2015). Moreover, the isolation of bird populations can exacerbate the differentiation among populations, even at small geographic distances (Sebastián‐González and Hart 2017).

As some acoustic traits are learned, a decrease in the flow of acoustic traits between two populations can lead to pronounced differences in species vocalizations (Campbell et al. 2010; Irwin et al. 2008; Podos and Warren 2007). For instance, if individuals within the same species fail to recognize each other due to substantial acoustic differences, this can expedite processes leading to speciation (e.g., Irwin et al. 2001). Furthermore, due to the relatively rapid nature of cultural transmission and change compared to genetic changes, birdsong can serve as an early indicator of the effects of habitat fragmentation on bird populations (Laiolo 2010; Pavlova, Amos, et al. 2012; Pavlova, Nevil, et al. 2012). Moreover, acoustic diversity can have significant implications even at the individual level, as individuals with a higher fitness and learning ability, as well as populations with a larger viability, have been found to exhibit more diverse and complex songs (Boogert et al. 2008; Laiolo et al. 2008; Crates et al. 2021).

In this study, we assess the variations in acoustic traits among three species of native Hawaiian honeycreepers on the Big Island of Hawaiʻi. There has been a gradual conversion of native forests into human settlements, agricultural lands, and pastures, particularly in the lowland regions of the island (Leopld and Hess 2017). Currently, the island comprises a heterogeneous landscape, with native forests remaining primarily at higher elevations, on the slopes of the five volcanoes of the island. Furthermore, Hawaiian honeycreepers exhibit high susceptibility to avian malaria (*Plasmodium relictum*), which primarily occurs in the low‐lying regions of the islands where the mosquito ( *Culex quinquefasciatus* ) acts as a vector for transmission of the disease (Van Riper III et al. 1986). Consequently, most Hawaiian honeycreepers are constrained to native forests above approximately 1300 m a.s.l., and the populations of these species across the volcanoes of the Big Island are currently fragmented and increasingly isolated, affecting gene flow (Van Riper III et al. 1986).

A previous study conducted on the same island but at a significantly smaller spatial distance (with a maximum distance of 5 km versus a 100 km distance among populations in the present study) investigated the acoustic diversity of these three bird species in forest patches (kīpuka) separated by relatively recent (< 10,000 years bp) lava flows. The findings indicated substantial acoustic variation among the species, with increased dissimilarity in vocalizations detected at greater distances for one of the species (Sebastián‐González and Hart 2017). Additionally, the study revealed a nested vocalization structure, where syllables in patches with lower syllable diversities were a subset of the syllables found in patches with higher syllable diversities across all species. Furthermore, one of the species (‘Apapane *Himatione sanguinea* ) exhibited distinct acoustic dialects. However, it is important to note that in the aforementioned study, populations were connected with frequent movements of individuals between kīpuka. In contrast, at the scale of the entire island, the movement of individuals among populations is restricted, and there is a heterogeneous landscape with different land cover types where these species live. In this study, we aim to (1) characterize the acoustic diversity of the three Hawaiian honeycreeper species on the Big Island of Hawaii, (2) compare the acoustic traits among bird populations of each species distributed across the island, and (3) determine whether the observed differences in acoustic traits among conspecific populations are primarily driven by cultural drift (i.e., isolation‐by‐distance) or landscape configuration. We hypothesize that dissimilarity among populations will be primarily influenced by the connectivity among populations rather than purely distance‐based factors.

## 2 Methods

### 2.1 Study Area and Species

**FIGURE 1:**
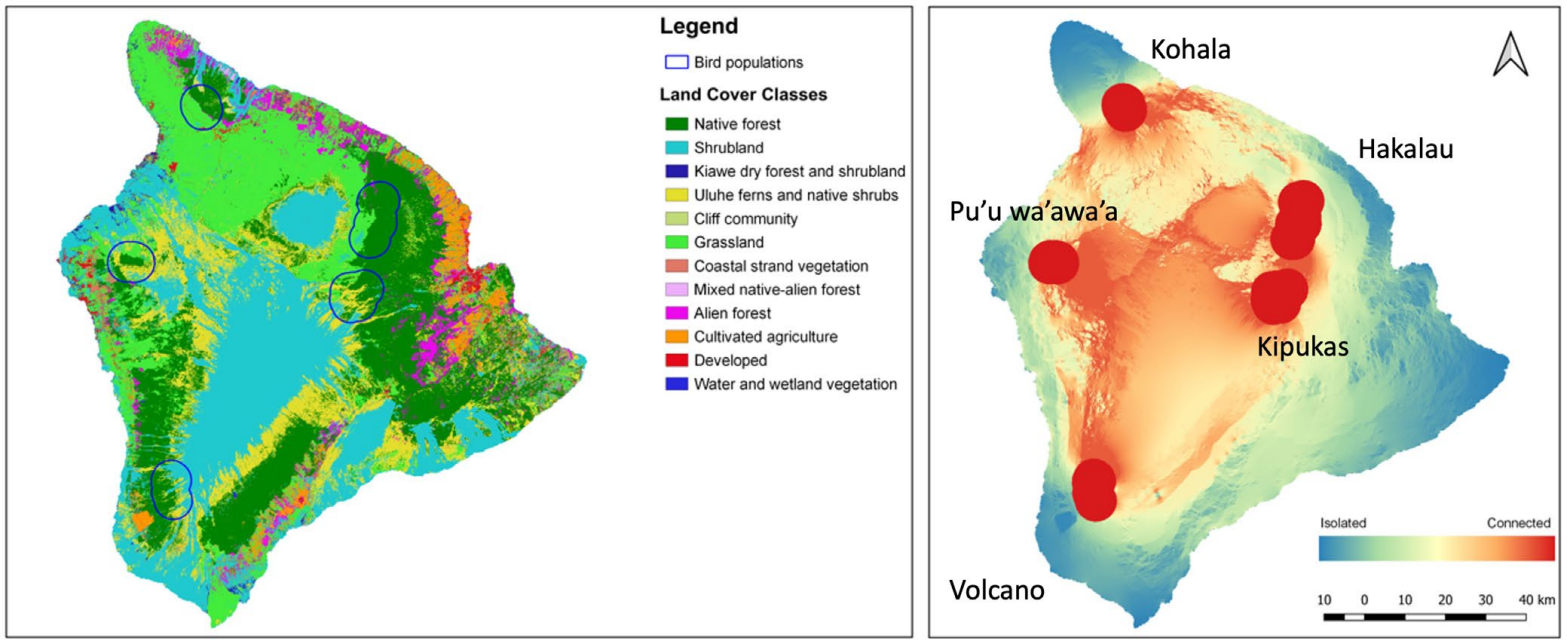
Map of the study area with the land cover classes used in this study (left) and the connectivity degree of the Hawai‘i Island for the ‘Amakihi (right). See Figure S1 for the same map for all the species. The blue circles (left map) and the red areas (right map) indicate the location of the five study sites.

Our study was performed on Hawai‘i Island (USA), the largest island in one of the most isolated archipelagos in the world (10,432 km^2^, Figure 1). The island is tropical (approx. 19°–20° latitude) and formed by five volcanoes, three of which are active, and there is a large gradient in terms of precipitation (range: 200–10,000 mm), mean annual temperature (range: 4°C–24°C) and altitude (range: 0–4207 m). The lowland regions of the island (ca. 0–1200 m) are mostly covered by non‐native vegetation, but at intermediate altitudes (ca. 1200–2500 m), where the study sites are located, there is a native canopy of ‘ōhi‘a ( *Metrosideros polymorpha* ) and koa ( *Acacia koa* ) tree forests (see Table S1 for the size of native forest in each of the five study areas). Most native Hawaiian birds are currently restricted to areas above approximately 1300 m elevation because human‐introduced diseases, such as avian malaria and pox, produce a high mortality in Hawaiian native birds, limiting their presence at low elevations, where mosquitoes are present (Fortini et al. 2015).

**FIGURE 2:**
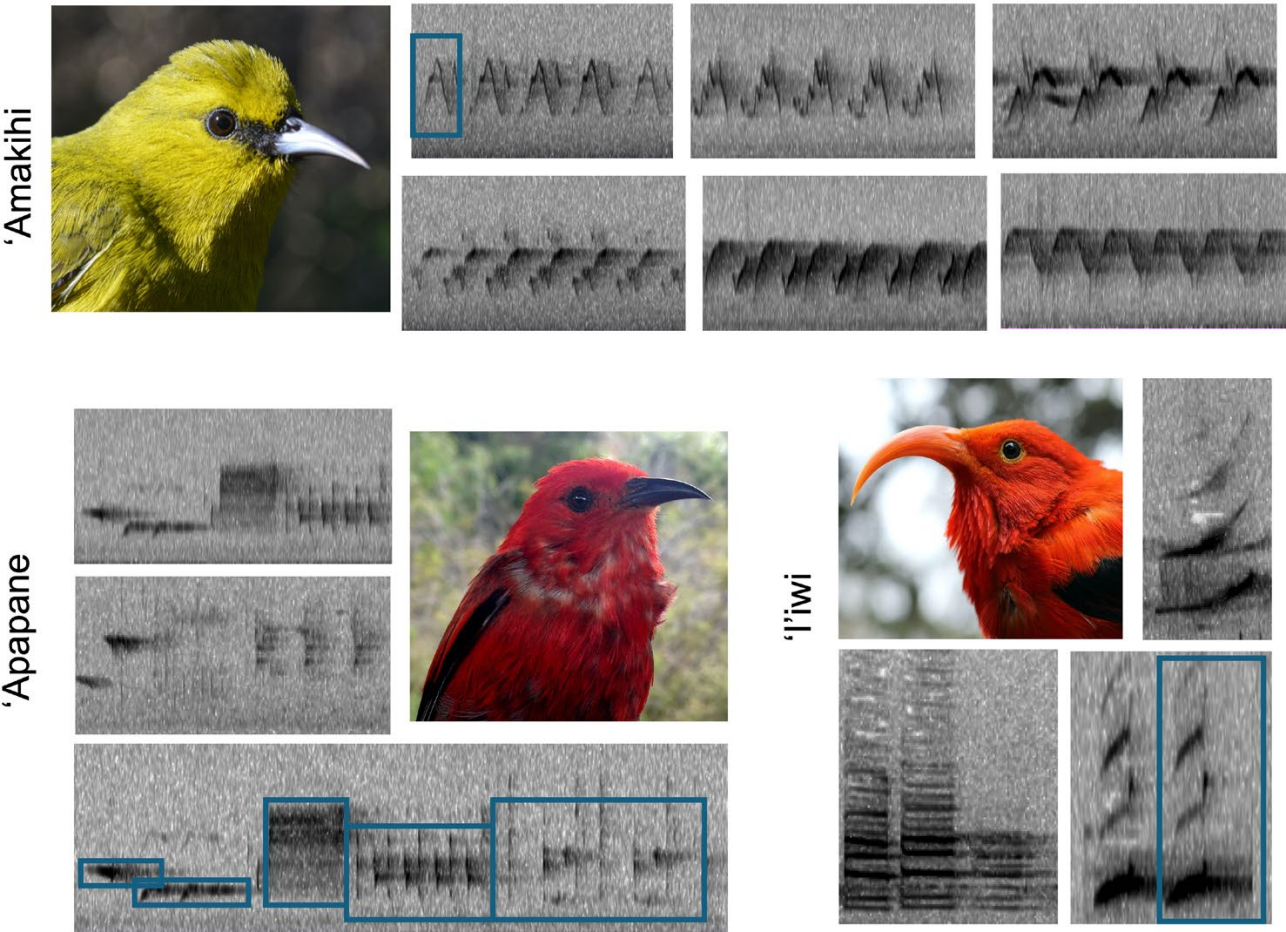
Spectrogram of characteristic songs of each of the three studies species. The *x*‐axis of the spectrograms represents time (s) and the *y*‐axis frequency (Hz). In one song per species, the different syllables that can be identified are marked in blue. Pictures: A. Tanimoto‐Johnson.

We studied three native Hawaiian forest bird species that are known to learn their vocalizations through cultural transmission (Beecher and Brenowitz 2005) and occur on the island at different densities (Table S1). The'Apapane ( *Himatione sanguinea* ) produces complex songs that are formed by several different syllables (Figure 2). They have a large repertoire; for example, 197 different syllables were detected in an area of less than 25 km^2^ (Sebastián‐González and Hart 2017). The'I‘iwi (*Drepanis coccinea*) has also been found to vocalize many different syllables (112 syllables in the same study above), but each song is often formed by 1–3 of them, although some long and very complex songs have also been detected. Finally, the song of the Hawai‘i'Amakihi ( *Chlorodrepanis virens* ) is a trill formed by the repetition of a single syllable. A more detailed description of the acoustic traits of these species can be found in Sebastián‐González and Hart (2017). The ‘Apapane and ‘I‘iwi are nectarivorous Hawaiian Honeycreepers (Fringillidae) whose diet relies mainly on the nectar of' ōhi'aflowers (Hart et al. 2011). The three species differ in their mobility patterns, with'Apapane being the most mobile of all the studied species (mean home range in a fragmented forest 42.4 ha vs. 181.7 ha in a continuous forest, Paxton et al. 2023), followed by the'I‘iwi (35.2 vs. 162.3 ha). The Hawai‘i'Amakihi is the least mobile of the three species (24.6 vs. 86.5 ha) and has a generalist diet based on nectar, fruits, and invertebrates (Pimm and Pimm 1982).

### 2.2 Study Design and Recordings

Within the Big Island of Hawaii, we selected 5 study areas across four volcanoes with similar native vegetation and where native Hawaiian birds are present (Figure 1). We spatially delimitated the position and size of the five landscapes based on the position of the recorders and the species “maximum distance moved” provided by Knowlton et al. (2017). Using this reference of maximum distance of movement, we created buffer zones surrounding each sound recorder and then merged these buffers when they spatially overlapped. As result, we obtained 5 polygons that delimited each study landscape (polygons with average area of 123 km^2^; SD: 25 km^2^; range 90–173 km^2^) (Figure 1). The mean distance among these areas is 61.8 km (SD: 22.3, range: 29.1–101.9, see Table S2 for details on pairwise distances). In each of these study areas, we placed 6 automatic recording units (SM2 Wildlife Acoustics) that were programmed to record for 5 min every 20 min from 6:00–11:00 00 (i.e., the hours of higher acoustic activity) and additionally from 15:00–16:00 to include also some recording out or the peak. Each recorder was actively recording approximately for a month, totaling 270 h of recordings per study area (90 min recorded per day × 30 days × 6 recorders). Recorders were separated by at least 1 km to ensure sample independence based on the typical movement patterns of the Hawaiian honeycreepers (Hart et al. 2011; Knowlton et al. 2017; Lindsey et al. 1998). Recordings were made in .WAV file format at a sampling rate of 44.1 kHz. Each recorder was equipped with an omnidirectional microphone (SMX‐II: Wildlife Acoustics) with a sensitivity of −35 dBV/pa and frequency response of 20–20,000 Hz. Recorders were placed on a tree 1.5 m high and were left in the field for approximately 2 weeks. We recorded in different dates between 2013 and 2016, always during the spring months (March–June, see Table S3 for exact recording dates at each study site).

### 2.3 Classification of Bird Acoustic Traits

From the available recordings, we excluded days with heavy rain or wind and then randomly selected four non‐consecutive days in each of the recording points, totaling 360 min of selected recordings for each recorder (90 min recorded per day × 4 days). Then, one of us (E.S.G.) visualized and listened to these 360 min of recordings, looking for syllables of the three species of interest. When characterizing ‘Amakihi and ‘Apapane, we only included songs, while‘I‘iwi vocalizations included both songs and calls. Calls are normally shorter and simpler than songs, and they are innate, while songs tend to be longer, more complex, and are learned. However, all ‘I‘iwi vocalizations are short and simple, and there is no information on which of its vocalizations are learned or innate, so it is not possible to differentiate among calls and songs for the ‘I‘iwi so far. However, most species (including Hawaiian Honeycreepers) only have a few calls, while the diversity of songs is large (see results), so including the calls for the ‘I‘iwi is not likely to affect our results. To classify the syllables, we used the range of values of the peak frequency, duration, spectrogram shape, and sound. Thus, for each new syllable described, we first identified its duration and frequency values. Any syllable differing in more than 1 kHz in frequency or more than 0.5 s in length would be considered a different syllable. By spectrogram shape, we mean the design of the syllable visualized in the spectrogram (Figure 2). We created an acoustic library by saving one example of each syllable that could help in their classification, as all the shapes could be visualized together and their sounds could be easily compared. The syllables were re‐evaluated several times during the classification process by E.S.G. to identify and delete possible duplicities and misclassifications. As an estimate, we re‐evaluated syllables when we finished classifying each recording point, so at least 30 times over the classification period. Syllables found only once through the entire study were excluded from further analyses. All the classifications were done using Raven Pro 1.4 software (K. Lisa Yang Center for Conservation Bioacoustics at the Cornell Lab of Ornithology 2024). We identified the presence/absence of each syllable type per species.

### 2.4 Acoustic Dissimilarity

We measured the dissimilarities in the repertoire (syllable identities) of each species among different locations using the Sørensen (1948) dissimilarity index (*D*_*kl*_), calculated as:

$$D_{\mathrm{kl}}=\left(S_{k}+S_{l}-2J\right)/\left(S_{k}+S_{l}\right)$$

where *S*_*k*_ is the number of syllables exclusive to a site *k*, *S*_*l*_ is the number of syllables exclusive to site *l*, and *J* is the number of syllables that are shared among the two sites. To do so, we first built a matrix *A* where each row *i* represented a study site and each column j represented a syllable. Each cell a*ij* was 1 when syllable *j* was present in site *i*, and 0 otherwise. We made the calculations using the *vegan* package (Oksanen et al. 2025) in R (R Development Core Team 2022).

### 2.5 Landscape Connectivity

We calculated the connectivity between populations using the geographical distance (shortest linear distance) and the cumulative landscape resistance (hereafter, resistance), based on a circuit theory, to evaluate how the level of isolation between pairs of bird populations affects the dissimilarity of acoustic repertories. To calculate connectivity as resistance, we used the Circuitscape computational approach (Anantharaman et al. 2020) that assesses the accumulated permeability/resistance of a pathway simulating the movement of the three studied birds between the five focal landscapes. In this approach, landscapes are represented as circuit boards, where each pixel in a raster depiction of the landscape is a resistor, and acoustic repertoire flow between any two subpopulations occurs via all possible chains of resistors linking them, not just along the single chain with the lowest sum of resistances (Dickson et al. 2018). This methodological approach helps to understand the effects of multiple dispersal pathways connecting samples by taking into account the land‐cover type (multidirectional movement), the species' likelihood of occurrence and displacement in each land cover, and the cumulative cost of multiple dispersal pathways. In addition, we adapted this approach to also include the bird's susceptibility to malaria based on its relationship with geographic elevation. These layers of information were obtained as follows.

Using the Hawai‘i GAP Analysis dataset (Gon III et al. 2006), we re‐categorized the different land covers in the Hawai‘i island into 12 final classes based on the most common land cover types of the island and the habitats used for our three focal bird species. The final land cover classes are: Alien forest, Coastal strand vegetation, Cultivated agriculture, Developed, Grassland, Kiawe dry forest and shrubland, Mixed native‐alien forest, Native forest, Cliff community, Shrubland, Uluhe ferns and native shrubs, Water & wetland vegetation (see Table S5 for their definition). Then, we used this land cover map (30 m resolution) to assess the species' likelihood of occurrence and displacement in each class. To determine this likelihood value, we asked nine ornithologists with extensive experience in Hawaiian birds (Table S4) to evaluate the probability of use (ranging between 0 and 1) of each land cover for each species. That is, we asked for the level of permeability/resistance of the bird's use of each land cover type as habitat or the bird's capacity for dispersion over each land cover type, ranging from 0 (not permeable, the bird will not cross neither use as habitat) to 1 (totally permeable, the bird uses as habitat and moves freely in the land cover type).

Further, as Hawaiian birds are highly affected by avian malaria (Samuel et al. 2015), we also defined a correction coefficient to the movement of these species due to malaria presence ranging from 0 (*malaria kills all the individuals of the population in this region*) to 1 (*malaria does not affect the species*). Therefore, we asked the experts to estimate a mortality coefficient in areas where malaria is present all the year (below 900 m of altitude), in the area where malaria is only present in the hottest months (altitude ranging 900 and 1500 m), and where malaria is usually absent (elevation larger than 1500 m.a.s.l.).

Then, we calculated the mean and SD of all these coefficients suggested by the nine ornithologists for each land cover and malaria risk (Table S6), permitting us to perform a sensitivity analysis within the Circuitscape analysis. To do this, we first produced raster grids with the potential conductance value (permeability/resistance of the habitat classes weighted by the mortality coefficient) using the Circuitscape computational tool (Anantharaman et al. 2020). Other input data into the model are grid rasters of each population between which conductance will be calculated, using a multidirectional movement framework. The cumulative conductance of the pairwise connectivity was calculated using the average, the minimum, and maximum standard deviation permeability/resistance coefficients obtained from the specialists' interviews. We finally obtained six raster grid maps of 30 m resolution (the same resolution of the land‐cover map) showing cumulative connectivity among the five landscapes for each of the three species and for the average, the minimum, and maximum standard deviation coefficients of resistance. Finally, we used the output of the pairwise mode of the Circuitscape analysis in the following statistical analyses.

### 2.6 Statistical Analyses

We compared the composition of the acoustic repertoire (i.e., the syllables vocalized) among the five study areas and for each species separately, by using Generalized Linear Models for Multivariate Abundance data using the *manyglm* function from the *mvabund* package (Wang et al. 2012). These models compare the number of individuals at the species level (in this study, the presence of a syllable) among different groups (in this study, study‐areas) by fitting one Generalized Linear Model (GLMs) for each species (i.e., syllable) and then using resampling‐based hypothesis testing to make community‐level inferences (Wang et al. 2012). Then, we ordered and visualized the differences in the vocalizations (i.e., the syllables vocalized) among the five study areas by means of a Principal Component Analysis (PCA) created using the *prcomp* function in R.

Finally, we tested if the pairwise dissimilarity (*β*‐diversity) of acoustic repertoires between two study areas increased with the linear shortest distance and the average accumulated resistance (landscapes connectivity) provided by the Circuitscape analysis. To do so, we used one‐predictor GLMs using a Gaussian distribution, where the *β*‐diversity was the response variable and either resistance or distance was the predictor. We selected models whose slope was significantly different from zero (i.e., *p* < 0.05). We repeated the analyses with the minimum and maximum accumulated resistances.

## 3 Results

### 3.1 Acoustic Diversity and Structure

We classified a total of 10,886 syllables from the three bird species at the five study sites, although we could not find any ‘I‘iwi at Volcano and thus this site was excluded for this species. We found between 52 and 215 different syllables per species (Tables 1 and Table S6). The three Hawaiian honeycreepers presented a large repertoire, as the number of syllables in a population ranged from 12 for ‘Amakihi to 105 for ‘Apapane. Also, a large proportion of syllables were only found in a single population. We also detected very high values of acoustic dissimilarity among study sites.

**TABLE 1:** Summary of song characteristics.

	‘Amakihi	‘Apapane	‘I‘iwi
Number of selections	1173	6647	3066
Total number of syllables	52	215	120
Range number of syllables	12–20	70–105	41–94
Exclusive syllables (%)	35 (67.3)	123 (57.2)	54 (45.0)
Average dissimilarity	0.70	0.55	0.44

*Note:* The number of selections made, total number of syllables, the range in the number of syllables in the different study areas, number (and percentage) of exclusive syllables (i.e., syllables that only appear in one area) and average dissimilarity (i.e., average value of all pairwise dissimilarities in repertoire among study areas) for each species. A summary of the song characteristics by study area can be found at Table S7.

### 3.2 Comparing Acoustic Traits Among Populations

In general, we found that the repertoire of the three species at each of the five studied populations was different, especially for the ‘Amakihi and ‘Apapane, except for a few comparisons (Table 2). In the case of the ‘I‘iwi, we found an overall non‐significant trend for the repertoires to be different (Table 2).

**TABLE 2:** Results of the generalized linear model for multivariate abundance data comparing the syllable composition among the different study areas for the three studied species.

Comparison	‘Amakihi		‘Apapane		‘I‘iwi	
	Coefficient	*p*	Coefficient	*p*	Coefficient	*p*
Hakalau vs. Kīpuka	35.01	*0.065*	170.2	*0.095*	211.7	*0.055*
Hakalau vs. Kohala	59.57	**0.045**	192.7	*0.080*	233.8	*0.050*
Hakalau vs. Volcano	50.63	*0.055*	269.6	**0.030**		
Hakalau vs. Pww	50.90	*0.055*	198.7	*0.080*	211.3	*0.055*
Kīpuka vs. Kohala	65.10	**0.030**	250.1	**0.030**	132.2	*0.080*
Kīpuka vs. Volcano	53.67	*0.055*	323.8	**0.020**		
Kīpuka vs. Pww	64.87	**0.030**	229.7	0.065	155.5	*0.080*
Kohala vs. Volcano	72.37	**0.015**	310.3	**0.020**		
Kohala vs. Pww	90.66	**0.005**	260.2	**0.030**	144.1	*0.080*
Volcano vs. Pww	93.44	**0.005**	299.0	**0.020**		

*Note:* Significant *p*‐values (< 0.05) are in bold and marginally significant (*p* < 0.1) are in italics.

Abbreviation: Pww: Pu’u wa’awa’a.

**FIGURE 3:**
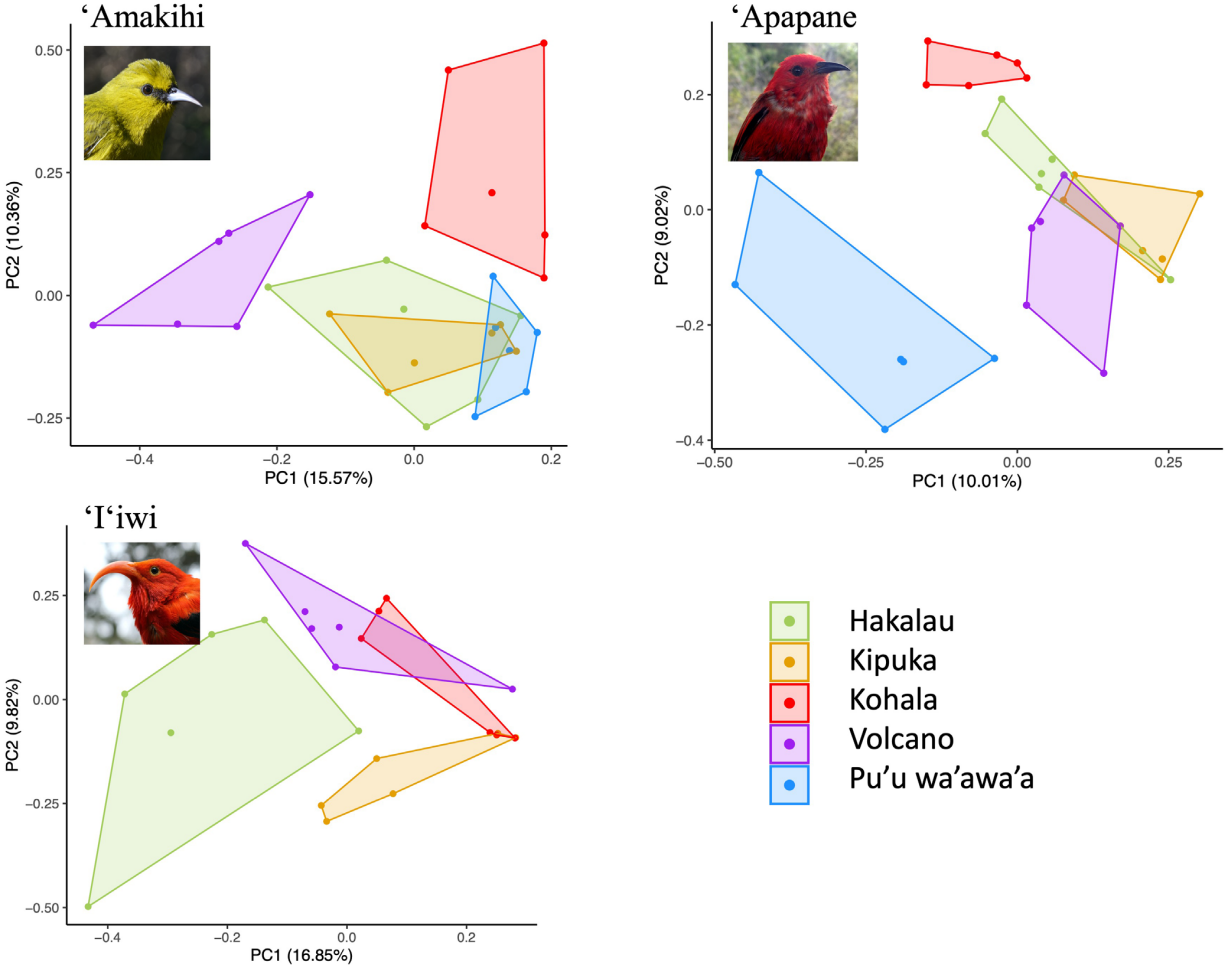
PCAs showing the differences in the acoustic composition (i.e., the syllables present) among the study areas for the three studied species. Pictures: A. Tanimoto‐Johnson.

Also, there was also a complete lack of overlap in the repertoires for some populations, including Kohala and Volcano for ‘Amakihi, Volcano and Pu'u wa'awa'a for ‘Apapane and among most study areas for ‘I‘iwi (Figure 3, explanatory power of the PCA ranging between 10% and 17% in axis 1 and between 9% and 10% in axis 2).

### 3.3 Effect of Distance and Isolation

The matrix of resistances shows that Hakalau and Kīpuka, with a minimum resistance value, are the best‐connected sites for all the 3 species (Figure 1 and Figure S1). Their closeness and the presence of contiguous native forest between the two areas could explain this value (Figure 1). On the other hand, Kohala is the most isolated population, presenting maximum resistance values with the other four study sites. These resistances would not be explained only by their distance, but also by the absence of permeable habitats between Kohala and all the others. These results are consistent with the map shown in Figure 1.

**FIGURE 4:**
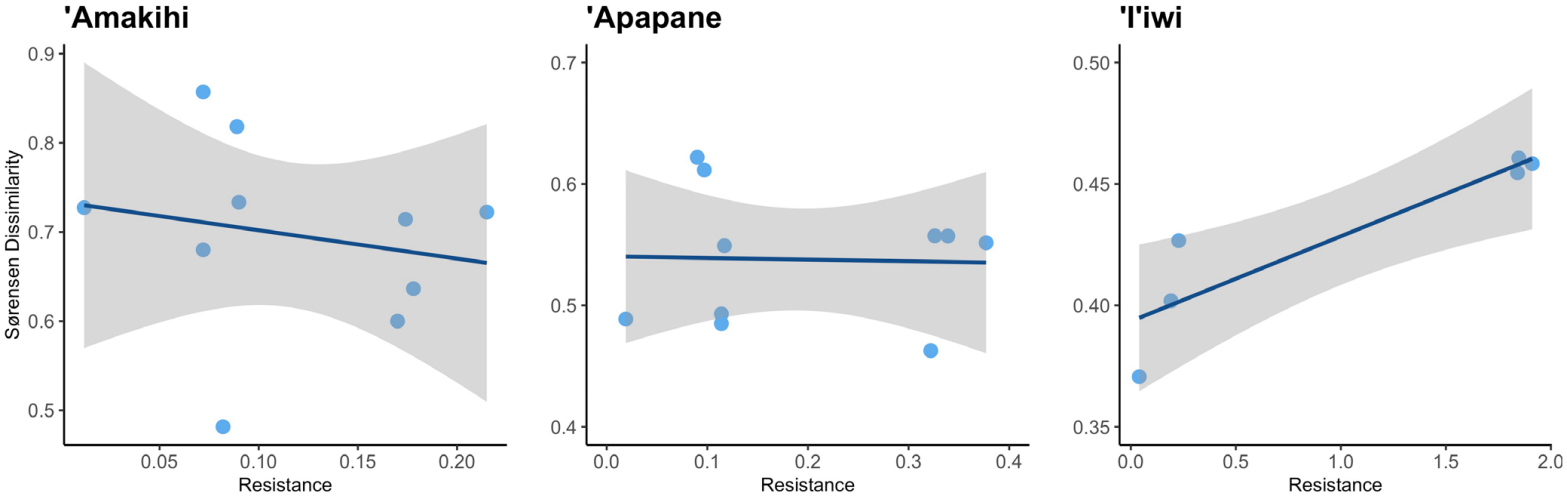
Relationship between the acoustic dissimilarity measured with the Sørensen dissimilarity index and the average resistance value calculated using Circuitscape. The resistance value represents the permeability/resistance of the landscape to the movement of each bird species. Each point is the value of the Sørensen dissimilarity among the acoustic traits of two study areas and the resistance value among them. The line is the linear regression, and the shaded area is its confidence interval.

The pairwise Sørensen dissimilarity in the repertoire was not related to the geographic distance for any of the three Hawaiian honeycreepers (Table 3). However, interestingly, the dissimilarity was significantly related to the degree of the isolation of the' I'iwipopulations (Figure 4). These results were consistent when using the maximum and minimum accumulated resistances (Table S8).

**TABLE 3:** GLMs relating the Sørensen dissimilarity with the resistance values and the linear distance.

Species	Predictor	Coefficient	*p*	*R* ^2^
‘Amakihi	Resistance	−0.007	0.959	0.001
	Distance	0.001	0.689	0.056
‘Apapane	Resistance	−0.301	0.610	0.034
	Distance	0.001	0.538	0.049
‘I‘iwi	Resistance	0.035	**0.014**	0.813
	Distance	−0.001	0.947	0.001

*Note:* Significant *p*‐values are shown in bold.

## 4 Discussion

In this study, we reveal a diverse syllable repertoire for three Hawaiian honeycreeper species. Moreover, we have found that there are differences in the acoustic repertoire of the species among the different study areas, suggesting the presence of dialects. We also provide evidence that human‐driven reduction of landscape connectivity has increased the isolation of the threatened ‘I'iwi populations and produced an increase in the dissimilarity of its learned acoustic traits. This study, along with the smaller scale one described at Sebastián‐González and Hart (2017), provides the first evidence that the ecological processes influencing the acoustic structure of Hawaiian honeycreepers can operate across varying spatial distances, ranging from meters to dozens of kilometers. As the effects of landscape connectivity on acoustic repertoire were not significant to the other two focal species, our study also highlights the variable effect of habitat fragmentation on the diversity of learned cultural traits, such as acoustic repertoire.

Our study describes a remarkably diverse acoustic repertoire in the three studied Hawaiian honeycreepers, with the ‘Apapane standing out by exhibiting over 200 syllables among the five populations. The combination of these syllables in different ways to form songs, especially for the ‘Apapane, further amplifies the range of vocalizations that can be heard on the Big Island of Hawaii. Moreover, there is a large proportion of syllables exclusive to single populations (ranging 45%–67%), which leads to the presence of substantial differences in the acoustic repertoire among populations for most of the species. Dialects can arise as a result of assortative mating (Wang et al. 2022), population isolation (Parker et al. 2012), or the adoption of neighboring songs (Nelson et al. 2004). Interestingly, a previous study suggested the existence of dialects for the'Apapane within spatial distances of less than 6 km (Sebastián‐González and Hart 2017). In that study, the formation of dialects was attributed to vocal matching by immigrant individuals with the local dialects, which is known as conformity (Morgan and Laland 2012). This aligns with the fact that‘Apapane individuals frequently move within the study site that was the focus of Sebastián‐González and Hart's paper but have very different repertoires in the different habitat patches (Knowlton et al. 2017; Smetzer et al. 2021). Likewise, our present study indicates that'Apapane individuals are more likely to move with less resistance across the landscape when compared to the other species. Finally, it is important to underline that, as both studies (this one and Sebastián‐González and Hart 2017) were done with automatic acoustic recorders, it was not possible to assess if the exclusive syllables were shared among different individuals within the population or if they were vocalized exclusively by a few individuals, so it is not possible to test if the exclusivity is a consequence of dialect formation or of individual variability.

However, documentation of individuals moving among the sites studied here is lacking. Thus, at the island scale, differences in the repertoire are more likely attributed to a limited exchange of individuals among populations and cultural drift. This is in agreement with the divergence in acoustic repertoires for these species occurring at smaller spatial distances. For instance, Ditzel (2023) observed an increasing dissimilarity of ‘Apapane songs within a two‐kilometer distance and entirely distinct songs after four kilometers. While Ditzel's study differed from ours in that he examined combinations of syllables, whereas we focused on individual syllables, it still reinforces the notion that the acoustic traits of these species undergo noticeable changes over smaller spatial distances. This is reinforced by the large dissimilarity values among populations found for the three species, especially for'Apapane and'Amakihi.

Numerous studies have explored how bird song traits change with distance and habitat fragmentation (Laiolo and Tella 2005; Pérez‐Granados et al. 2016; Hart et al. 2018). However, habitat fragmentation per se does not necessarily have negative effects on species (e.g., Fahrig 2017) and the impact of landscape connectivity following fragmentation in acoustic traits remains less understood (Pavlova, Amos, et al. 2012; Pavlova, Nevil, et al. 2012). Our findings indicate that, in fragmented landscapes, habitat connectivity may serve as a more accurate predictor of acoustic dissimilarity than geographic distance for some species such as the ‘I'iwi, a threatened Hawaiian honeycreeper. Decreased song sharing among bird populations is observed when barriers hinder individual' movement between populations (e.g., Camacho‐Alpízar et al. 2018). In our study area, the highest acoustic dissimilarity was found in the most distant and less connected population for the ‘I'iwi, which is the species most sensitive to avian malaria and thus to crossing the lowland landscape (Liao et al. 2017). Interestingly, the ‘I'iwi also exhibited increased acoustic dissimilarity with distance over a smaller spatial distance in a naturally fragmented landscape (Sebastián‐González and Hart 2017). However, acoustic dissimilarity was not related to shortest distance or landscape connectivity for the other two studied species (‘Apapane and ‘Amakihi). Similarly, Goretskaia et al. (2018) found no influence of landscape connectivity on song similarity at a similar spatial distance in an Australian landscape. Consequently, future research should expand the investigation of the effects of habitat fragmentation and loss by considering connectivity among habitat patches, as well as other potential drivers such as fragment and population size, while recognizing that acoustic differences among habitat fragments may occur across various spatial scales.

Due to human‐driven reduction in landscape connectivity and the expected increase in habitat fragmentation and loss over time, the acoustic differences among some Hawaiian Honeycreeper populations may escalate. Since these species acquire acoustic traits through learning from conspecifics (Petkov and Jarvis 2012), the significant acoustic dissimilarity among populations suggests limited exchange of individuals. This, coupled with the low densities described for some populations (see Table S1), indicates increased vulnerability to catastrophic events (Paxton et al. 2019). Furthermore, recent studies highlight the importance of preserving not only species diversity but also the diversity in animal culture (Brakes et al. 2021; Cordero‐Rivera 2017; Backhouse et al. 2023). Therefore, based on studies that have found that Hawaiian honeycreepers tend to move more in continuous than in fragmented habitats (Paxton et al. 2023), measures to enhance connectivity among populations within the Hawaiʻi Island are crucially needed.

We chose a connectivity model based on circuit theory to test our hypotheses because it assumes a multidirectional movement pattern for the three bird species with a good capacity for habitat exploration. Other models such as least‐cost models assume fixed routes established over generations, whereas these birds have greater flexibility of movement. As shown by McClure et al. (2016), Circuitscape performs better than least‐cost paths in cases such as ours, as it reflects random exploration of the landscape, as opposed to the rigidity of other types of deep‐seated migratory routes.

### 4.1 Caveats

Despite the large sample size in terms of acoustic recordings and study areas used here, there are some limitations on our study. First, the recordings at the different sites were not all done at the same time, and they were conducted over a 4‐year period. It is important to note that certain areas had limited accessibility, which hindered the placement and retrieval of the automatic recorders. Nevertheless, we do not anticipate substantial differences in the acoustic traits over the recording period, as the lifespan of these species exceeds the duration of the study (Billerman et al. 2022). Also, Ditzel (2023) compared the repertoire of one of the species (the ‘Apapane) over 10 years and found that it had remained similar over that time. Although the period of the recordings and the land cover map are not the same, we also do not expect substantial changes in our results when using different land cover maps, as bird song learning may occur in a wide temporal range and depend on past or historical dispersal processes. However, future studies may explore the potential role of a temporal debt on the effect of habitat degradation or isolation on losses of bird vocalization. Second, the resistance model was based on expert's knowledge and not on empirical data. Getting enough information to build this model with field data would not be practical. However, even if there are some biases in the expert's assessments, averaging the values of several of them has been found to be a good approach when field data is missing, as these show a fair alignment with real data (Bennett et al. 2023). Third, in constructing the Circuitscape model, we employed the maximum observed movement distances in a fragmented landscape as reported by Knowlton et al. (2017) to define all the population areas. Other studies have identified larger movement distances for some of the studied species in a more continuous forest (Smetzer et al. 2021). We opted for the distances from Knowlton et al. (2017) due to the unavailability of data for one of the studied species at Smetzer's study. Additionally, we anticipate that changes in the buffer sizes will not significantly impact our conclusions, as we utilized the same buffer size across all study sites, ensuring comparability of results. It is worth noting that the maximum possible changes in buffer size are 3 km, whereas the distances among populations range from 30 to 102 km. Fourth, we must recognize that syllables may have been defined slightly differently for ‘I'iwi compared to the other species. Lastly, we would also like to recognize that, as we used automatic sound recorders in this study, we cannot be sure of the number of different individuals recorded over the study period at each study area. However, the six different study points are separated enough so that samples are independent. Moreover, the individuals of these species move often through the landscape (Paxton et al. 2023), and it is common to see several individuals of the same species singing at the same point and at the same time (E.S.G. & P.J.H., personal observations).

## 5 Conclusions

In conclusion, this study provides significant insights into the acoustic diversity and population structure of three Hawaiian honeycreeper species, particularly the ‘I'iwi, ‘Apapane, and ‘Amakihi. Our findings reveal a rich repertoire of syllables that vary between populations, suggesting the presence of dialects and a notable degree of acoustic diversity even over small spatial scales. The ‘Apapane stands out with its large syllable count, showcasing its potential for vocal flexibility and song variability. Additionally, we found evidence that human‐driven habitat fragmentation has exacerbated isolation among honeycreeper populations, particularly for the threatened ‘I'iwi, leading to increased acoustic dissimilarity.

This study underscores the importance of considering habitat connectivity when assessing the impact of fragmentation on cultural traits such as bird song. While geographic distance has traditionally been used to explain acoustic dissimilarity, our findings suggest that landscape connectivity may serve as a better predictor in fragmented environments, especially for species with limited dispersal abilities. As environmental pressures continue to mount, maintaining both species diversity and the cultural traits they exhibit will be crucial for the long‐term survival of Hawaiian honeycreepers.

## Author Contributions

**Esther Sebastián‐González:** conceptualization (equal), data curation (lead), formal analysis (equal), methodology (equal), writing – original draft (lead). **Jomar M. Barbosa:** data curation (equal), formal analysis (equal), methodology (equal), writing – review and editing (equal). **Pablo Montoya‐Bernabeu:** data curation (supporting), formal analysis (equal), methodology (equal), writing – review and editing (equal). **Patrick J. Hart:** conceptualization (equal), funding acquisition (lead), methodology (equal), project administration (lead), writing – review and editing (equal).

## Conflicts of Interest

The authors declare no conflicts of interest.

## Supporting information


**Data S1:** ece371919‐sup‐0001‐Supinfo.docx.

## Data Availability

The data that support the findings of this study are openly available in Figshare at https://figshare.com/s/a25cd2699a345ca98a04.

## References

[ece371919-bib-0001] Anantharaman, R. , K. Hall , V. B. Shah , and A. Edelman . 2020. “Circuitscape in Julia: High Performance Connectivity Modelling to Support Conservation Decisions.” Proceedings of the Julia Conferences 1, no. 1: 1–6.

[ece371919-bib-0002] Backhouse, F. , J. A. Welbergen , R. D. Magrath , and A. H. Dalziell . 2023. “Depleted Cultural Richness of an Avian Vocal Mimic in Fragmented Habitat.” Diversity and Distributions 29, no. 1: 109–122.

[ece371919-bib-0003] Beecher, M. D. , and E. A. Brenowitz . 2005. “Functional Aspects of Song Learning in Songbirds.” Trends in Ecology & Evolution 20: 143–149. 16701358 10.1016/j.tree.2005.01.004

[ece371919-bib-0004] Bełcik, M. , M. Lenda , T. Amano , and P. Skórka . 2020. “Different Response of the Taxonomic, Phylogenetic and Functional Diversity of Birds to Forest Fragmentation.” Scientific Reports 10, no. 1: 20320. 33230280 10.1038/s41598-020-76917-2 PMC7683534

[ece371919-bib-0005] Bennett, A. F. , A. Haslem , M. White , T. Hollings , and J. R. Thomson . 2023. “How Expert Are ‘Experts’? Comparing Expert Predictions and Empirical Data on the Use of Farmland Restoration Sites by Birds.” Biological Conservation 282: 110018.

[ece371919-bib-0006] Billerman, S. M. , B. K. Keeney , P. G. Rodewald , and T. S. Schulenberg , eds. 2022. Birds of the World. Cornell Laboratory of Ornithology. https://birdsoftheworld.org/bow/home.

[ece371919-bib-0007] Boogert, N. J. , L. A. Giraldeau , and L. Lefebvre . 2008. “Song Complexity Correlates With Learning Ability in Zebra Finch Males.” Animal Behaviour 76, no. 5: 1735–1741.

[ece371919-bib-0008] Brakes, P. , E. L. Carroll , S. R. Dall , et al. 2021. “A Deepening Understanding of Animal Culture Suggests Lessons for Conservation.” Proceedings of the Royal Society B: Biological Sciences 288, no. 1949: 20202718. 10.1098/rspb.2020.2718 PMC8059593 33878919

[ece371919-bib-0009] Camacho‐Alpízar, A. , E. J. Fuchs , and G. Barrantes . 2018. “Effect of Barriers and Distance on Song, Genetic, and Morphological Divergence in the Highland Endemic Timberline Wren (*Thryorchilus browni*, Troglodytidae).” PLoS One 13, no. 12: e0209508. 10.1371/journal.,pone.0209508. 30571751 PMC6301610

[ece371919-bib-0010] Campbell, P. , B. Pasch , J. L. Pino , O. L. Crino , M. Phillips , and S. M. Phelps . 2010. “Geographic Variation in the Songs of Neotropical Singing Mice: Testing the Relative Importance of Drift and Local Adaptation.” Evolution 64, no. 7: 1955–1972. 20148958 10.1111/j.1558-5646.2010.00962.x

[ece371919-bib-0011] Cordero‐Rivera, A. 2017. “Behavioral Diversity (Ethodiversity): A Neglected Level in the Study of Biodiversity.” Frontiers in Ecology and Evolution 5: 7.

[ece371919-bib-0012] Crates, R. , N. Langmore , L. Ranjard , et al. 2021. “Loss of Vocal Culture and Fitness Costs in a Critically Endangered Songbird.” Proceedings of the Royal Society B 288, no. 1947: 20210225. 33726592 10.1098/rspb.2021.0225 PMC8059949

[ece371919-bib-0013] Crooks, K. R. , C. L. Burdett , D. M. Theobald , et al. 2017. “Quantification of Habitat Fragmentation Reveals Extinction Risk in Terrestrial Mammals.” Proceedings of the National Academy of Sciences of the United States of America 114, no. 29: 7635–7640. 28673992 10.1073/pnas.1705769114 PMC5530695

[ece371919-bib-0014] Dickson, B. G. , C. M. Albano , R. Anantharaman , et al. 2018. “Circuit‐Theory Applications to Connectivity Science and Conservation.” Conservation Biology 33: 239–249. 30311266 10.1111/cobi.13230 PMC6727660

[ece371919-bib-0015] Ding, Z. , K. J. Feeley , Y. Wang , R. J. Pakeman , and P. Ding . 2013. “Patterns of Bird Functional Diversity on Land‐Bridge Island Fragments.” Journal of Animal Ecology 82, no. 4: 781–790. 23506201 10.1111/1365-2656.12046

[ece371919-bib-0016] Ditzel, P. C. 2023. “Vocal Behavior and Dialect Distribution in an Endemic Hawaiian Songbird Species ( *Himatione sanguinea* )” Master's Thesis Dissertation. Freie Universität, Berlin, Germany.

[ece371919-bib-0018] Fahrig, L. 2003. “Effects of Habitat Fragmentation on Biodiversity.” Annual Review of Ecology, Evolution, and Systematics 34, no. 1: 487–515.

[ece371919-bib-0019] Fahrig, L. 2017. “Ecological Responses to Habitat Fragmentation Per Se.” Annual Review of Ecology, Evolution, and Systematics 48, no. 1: 1–23.

[ece371919-bib-0020] Fortini, L. B. , A. E. Vorsino , F. A. Amidon , E. H. Paxton , and J. D. Jacobi . 2015. “Large‐Scale Range Collapse of Hawaiian Forest Birds Under Climate Change and the Need 21st Century Conservation Options.” PLoS One 10, no. 10: e0140389. 26509270 10.1371/journal.pone.0140389 PMC4625087

[ece371919-bib-0021] Galán‐Acedo, C. , L. Fahrig , F. Riva , and T. Schulz . 2024. “Positive Effects of Fragmentation Per Se on the Most Iconic Metapopulation.” Conservation Letters 17: e13017.

[ece371919-bib-0022] Gon, S. M., III , A. Allison , R. J. Cannarella , et al. 2006. A GAP Analysis of Hawaii—Final Report: U.S. Geological Survey, Research Corporation of the University of Hawaiʻi 163 p, Plus Maps and Appendixes.

[ece371919-bib-0023] Goretskaia, M. I. , I. R. Beme , D. V. Popova , et al. 2018. “Song Parameters of the Fuscous Honeyeater *Lichenostomus fuscus* Correlate With Habitat Characteristics in Fragmented Landscapes.” Journal of Avian Biology 49, no. 2: 1493.

[ece371919-bib-0024] Haddad, N. M. , L. A. Brudvig , J. Clobert , et al. 2015. “Habitat Fragmentation and Its Lasting Impact on Earth's Ecosystems.” Science Advances 1, no. 2: e1500052. 10.1126/sciadv.1500052. 26601154 PMC4643828

[ece371919-bib-0025] Hart, P. J. , E. Sebastián‐González , A. Tanimoto , et al. 2018. “Birdsong Characteristics Are Related to Fragment Size in a Neotropical Forest.” Animal Behaviour 137: 45–52.

[ece371919-bib-0026] Hart, P. J. , B. L. Woodworth , R. J. Camp , et al. 2011. “Temporal Variation in Bird and Resource Abundance Across an Elevational Gradient in Hawaii.” Auk 128: 113–126.

[ece371919-bib-0027] Irwin, D. E. , S. Bensch , and T. D. Price . 2001. “Speciation in a Ring.” Nature 409: 333–337. 11201740 10.1038/35053059

[ece371919-bib-0028] Irwin, D. E. , M. P. Thimgan , and J. H. Irwin . 2008. “Call Divergence Is Correlated With Geographic and Genetic Distance in Greenish Warblers ( *Phylloscopus trochiloides* ): A Strong Role for Stochasticity in Signal Evolution?” Journal of Evolutionary Biology 21, no. 2: 435–448. 18205774 10.1111/j.1420-9101.2007.01499.x

[ece371919-bib-0029] K. Lisa Yang Center for Conservation Bioacoustics at the Cornell Lab of Ornithology . 2024. Raven Pro: Interactive Sound Analysis Software (Version 1.6.5) [Computer Software]. Cornell Lab of Ornithology. https://www.ravensoundsoftware.com/.

[ece371919-bib-0030] Knowlton, J. L. , D. J. Flaspohler , E. H. Paxton , et al. 2017. “Movements of Four Native Hawaiian Birds Across a Naturally Fragmented Landscape.” Early View in Journal of Avian Biology 8: 921–931. 10.1111/jav.00924.

[ece371919-bib-0031] Laiolo, P. 2010. “The Emerging Significance of Bioacoustics in Animal Species Conservation.” Biological Conservation 143, no. 7: 1635–1645.

[ece371919-bib-0032] Laiolo, P. , and J. L. Tella . 2005. “Habitat Fragmentation Affects Culture Transmission: Patterns of Song Matching in Dupont's Lark.” Journal of Applied Ecology 42: 1183–1193.

[ece371919-bib-0033] Laiolo, P. , M. Vogeli , D. Serrano , and J. L. Tella . 2008. “Song Diversity Predicts the Viability of Fragmented Bird Populations.” PLoS One 3: e1822. 18350158 10.1371/journal.pone.0001822 PMC2266806

[ece371919-bib-0034] Laurance, W. F. 2014. “Contemporary Drivers of Habitat Fragmentation.” In Global Forest Fragmentation, 20–27. CABI.

[ece371919-bib-0035] Leopld, C. R. , and S. C. Hess . 2017. “Conversion of Native Terrestrial Ecosystems in Hawaiʻi to Novel Grazing Systems: A Review.” Biological Invasions 19: 161–177.

[ece371919-bib-0063] Liao, W. , C. T. Atkinson , D. A. LaPointe , and M. D. Samuel . 2017. “Mitigating Future Avian Malaria Threats to Hawaiian Forest Birds From Climate Change.” PLoS One 12, no. 1: e0168880. 28060848 10.1371/journal.pone.0168880 PMC5218566

[ece371919-bib-0036] Lindsey, G. D. , E. A. VanderWerf , H. Baker , and P. E. Baker . 1998. “Hawai‘i Amakihi ( *Hemignathus virens* ).” In The Birds of North America, edited by A. Poole . Cornell Lab of Ornithology.

[ece371919-bib-0037] Liu, J. , D. A. Coomes , L. Gibson , et al. 2019. “Forest Fragmentation in China and Its Effect on Biodiversity.” Biological Reviews 94, no. 5: 1636–1657. 31058438 10.1111/brv.12519

[ece371919-bib-0038] MacArthur, R. H. , and E. O. Wilson . 1967. The Theory of Island Biogeography. Vol. 1. Princeton university press.

[ece371919-bib-0039] McClure, M. L. , A. J. Hansen , and R. M. Inman . 2016. “Connecting Models to Movements: Testing Connectivity Model Predictions Against Empirical Migration and Dispersal Data.” Landscape Ecology 31: 1419–1432. 10.1007/s10980-016-0347-0.

[ece371919-bib-0040] Morgan, T. J. H. , and K. N. Laland . 2012. “The Biological Bases of Conformity.” Frontiers in Neuroscience 6: 1–7. 22712006 10.3389/fnins.2012.00087 PMC3375089

[ece371919-bib-0041] Nelson, D. A. , K. I. Hallberg , and J. A. Soha . 2004. “Cultural Evolution of Puget Sound White‐Crowned Sparrow Song Dialects.” Ethology 110, no. 11: 879–908.

[ece371919-bib-0062] Oksanen, J. , G. Simpson , F. Blanchet , et al. 2025. vegan: Community Ecology Package. R Package Version 2.8‐0. https://vegandevs.github.io/vegan/.

[ece371919-bib-0042] Parker, K. A. , M. J. Anderson , P. F. Jenkins , and D. H. Brunton . 2012. “The Effects of Translocation‐Induced Isolation and Fragmentation on the Cultural Evolution of Bird Song.” Ecology Letters 15: 778–785. 22590997 10.1111/j.1461-0248.2012.01797.x

[ece371919-bib-0043] Pavlova, A. , J. N. Amos , M. I. Goretskaia , et al. 2012. “Genes and Song: Genetic and Social Connections in Fragmented Habitat in a Woodland Bird With Limited Dispersal.” Ecology 93: 1717–1727. 22919917 10.1890/11-1891.1

[ece371919-bib-0044] Pavlova, A. , J. Nevil , M. A. Gorestskaia , et al. 2012. “Genes and Song: Genetic and Social Connections in Fragmented Habitat in a Woodland Bird With Limited Dispersal.” Ecology 9, no. 7: 1717–1727. 10.1890/11-1891.1 22919917

[ece371919-bib-0045] Paxton, K. L. , E. Sebastián‐González , J. M. Hite , L. H. Crampton , D. Kuhn , and P. J. Hart . 2019. “Loss of Cultural Song Diversity and the Convergence of Songs in a Declining Hawaiian Forest Bird Community.” Royal Society Open Science 6, no. 8: 190719. 31598249 10.1098/rsos.190719 PMC6731710

[ece371919-bib-0046] Paxton, K. L. , J. R. Smetzer , P. J. Hart , M. J. Anderson , and E. H. Paxton . 2023. “Landscape Configuration Alters Movement Behavior and Space‐Use of a Hawaiian Forest Bird Community.” Journal of Avian Biology 2024: e03117.

[ece371919-bib-0047] Pérez‐Granados, C. , T. Osiejuk , and G. M. López‐Iborra . 2016. “Habitat Fragmentation Effects and Variations in Repertoire Size and Degree of Song Sharing Among Close Dupont's Lark *Chersophilus duponti* Populations.” Journal of Ornithology 157: 471–482.

[ece371919-bib-0048] Petkov, C. I. , and E. D. Jarvis . 2012. “Birds, Primates, and Spoken Language Origins: Behavioral Phenotypes and Neurobiological Substrates.” Frontiers in Evolutionary Neuroscience 4: 12. 22912615 10.3389/fnevo.2012.00012 PMC3419981

[ece371919-bib-0049] Pimm, S. L. , and J. W. Pimm . 1982. “Resource Use Competition and Resource Availability in Hawaiian Honeycreepers.” Ecology 63: 1468–1480.

[ece371919-bib-0050] Podos, J. , and P. S. Warren . 2007. “The Evolution of Geographic Variation in Birdsong.” Advances in the Study of Behavior 37: 403–458.

[ece371919-bib-0051] Potvin, D. A. , and S. M. Clegg . 2015. “The Relative Roles of Cultural Drift and Acoustic Adaptation in Shaping Syllable Repertoires of Island Bird Populations Change With Time Since Colonization.” Evolution 69, no. 2: 368–380. 25496402 10.1111/evo.12573

[ece371919-bib-0052] Prugh, L. R. , K. E. Hodges , A. R. Sinclair , and J. S. Brashares . 2008. “Effect of Habitat Area and Isolation on Fragmented Animal Populations.” Proceedings of the National Academy of Sciences of the United States of America 105, no. 52: 20770–20775. 19073931 10.1073/pnas.0806080105 PMC2634894

[ece371919-bib-0064] R Core Team . 2022. R: A Language and Environment for Statistical Computing. R Foundation for Statistical Computing. https://www.R‐project.org/.

[ece371919-bib-0053] Samuel, M. D. , B. L. Woodworth , C. T. Atkinson , P. J. Hart , and D. A. LaPointe . 2015. “Avian Malaria in Hawaiian Forest Birds: Infection and Population Impacts Across Species and Elevations.” Ecosphere 6, no. 6: 1–21.

[ece371919-bib-0054] Schlaepfer, D. R. , B. Braschler , H. P. Rusterholz , and B. Baur . 2018. “Genetic Effects of Anthropogenic Habitat Fragmentation on Remnant Animal and Plant Populations: A Meta‐Analysis.” Ecosphere 9, no. 10: e02488.

[ece371919-bib-0055] Sebastián‐González, E. , and P. J. Hart . 2017. “Birdsong Syllable Diversity in a Habitat Landscape Depends on Landscape and Species Characteristics.” Oikos 126, no. 10: 1511–1521.

[ece371919-bib-0056] Smetzer, J. R. , K. L. Paxton , and E. H. Paxton . 2021. “Individual and Seasonal Variation in the Movement Behavior of Two Tropical Nectarivorous Birds.” Movement Ecology 9, no. 1: 1–15. 34233764 10.1186/s40462-021-00275-5 PMC8264974

[ece371919-bib-0057] Sørensen, T. 1948. “A Method of Establishing Groups of Equal Amplitude in Plant Sociology Based on Similarity of Species Content and Its Application to Analyses of the Vegetation on Danish Commons.” Biologiske Skrifter 5: 1–34.

[ece371919-bib-0058] Valente, J. J. , D. G. Gannon , J. Hightower , et al. 2023. “Toward Conciliation in the Habitat Fragmentation and Biodiversity Debate.” Landscape Ecology 38, no. 11: 2717–2730.

[ece371919-bib-0059] Van Riper, C., III , S. G. Riper , M. L. Goff , and M. Laird . 1986. “The Epizootiology and Ecological Significance of Malaria in Hawaiian Land Birds.” Ecological Monographs 56, no. 4: 327–344.

[ece371919-bib-0060] Wang, D. , W. Forstmeier , D. R. Farine , et al. 2022. “Machine Learning Reveals Cryptic Dialects That Explain Mate Choice in a Songbird.” Nature Communications 13, no. 1: 1630. 10.1038/s41467-022-28881-w PMC8960899 35347115

[ece371919-bib-0061] Wang, Y. I. , U. Naumann , S. T. Wright , and D. I. Warton . 2012. “Mvabund–an R Package for Model‐Based Analysis of Multivariate Abundance Data.” Methods in Ecology and Evolution 3, no. 3: 471–474.

