# Supplementary material for: Acoustic Traits of Three Hawaiian Honeycreepers in a Fragmented Landscape
Source: Ecol Evol. 2025 Aug 11;15(8):e71919. doi: 10.1002/ece3.71919 (PMC12336420; doi:10.1002/ece3.71919)

**Acoustic diversity and meme repertoire of three Hawaiian Honeycreepers in a fragmented landscape**

Esther Sebastián-González, Jomar M Barbosa, Pablo Montoya and Patrick J. Hart

**Supplementary material**

**Table S1.** Mean density (individuals per hectare) of each species at each study area and total extension of native forest in each study area. We also include the information on the publication used as source of the data and on the year when the density was calculated.

| **Site** | **‘Amakihi** | **‘Apapane** | **‘I‘iwi** | **Year** | **Reference** |
| --- | --- | --- | --- | --- | --- |
| Hakalau | 10.70 | 14.43 | 17.06 | 2015 | Kendall et al. 2022 |
| Kipuka | 12.30 | 39.80 | 8.00 | 2012 | Kovach 2012 |
| Kohala | 14.91 | 18.57 | 1.34 | 2017 | Burnett et al. 2021 |
| Pu’u wa’a wa’a | 21.89 | 10.38 | 1.96 | 2003 | Camp et al. 2009 |
| Volcano | 7.32 | 16.62 | 0.52 | 2015 | Judge et al. 2017 |

**Table S2.** Pairwise distances among the center of the five study areas in kilometers.

| **Site 1** | **Site 2** | **Distance (km)** |
| --- | --- | --- |
| Hakalau | Kipuka | 29.14 |
| Hakalau | Kohala | 45.54 |
| Hakalau | Volcano | 94.40 |
| Hakalau | Pu’u wa’a wa’a | 62.62 |
| Kipuka | Kohala | 61.59 |
| Kipuka | Volcano | 68.12 |
| Kipuka | Pu’u wa’a wa’a | 52.58 |
| Kohala | Volcano | 101.9 |
| Kohala | Pu’u wa’a wa’a | 43.94 |
| Volcano | Pu’u wa’a wa’a | 58.29 |

**Table S3.** Study areas and their recording months.

| **Location** | **Recording dates** | **Recording months** |
| --- | --- | --- |
| Hakalau | 2015-2016 | March, April |
| Kipuka | 2014 | June, September |
| Kohala | 2015-2016 | April-June |
| Volcano | 2015-2016 | March-May |
| Pu’u wa’a wa’a | 2013 | April-May |

**Table S4.** List of experts that gave information on the permeability of the landscape for the Hawaiian birds.

| **Name** | **Affiliation** |
| --- | --- |
| P.J. Hart | Full professor. University of Hawaii at Hilo |
| J.D. Jacobi | Biologist. U. S. Geological Survey |
| C. Farmer | American Bird Conservancy |
| E. Paxton | Research ecologist. U. S. Geological Survey |
| E. VanderWerf | Pacific Rim Conservation |
| A. Wang | Department of Land and Natural Sciences |
| P. Banko | Research Wildlife Biologist. U. S. Geological Survey |
| S. Kendall | U. S. Fish and Wildlife Service |
| S. Judge | Terrestrial Ecologist. Natural Park Service |

**Table S5.** List of habitat classes included in the resistance analysis and their description.

| **Habitat class** | **Description** |
| --- | --- |
| Alien forest | Mixed, typically dense canopies of alien tree species, often plantation forest plantings, with dominants including, but not limited to: *Eucalyptus, Casuarina, Paraserianthes, Araucaria, Fraxinus, Melaleuca, Psidium,* and *Grevillea* spp. At lowland and montane, wet-mesic to mesic settings, often at the lower elevation edge of forest reserves. |
| Cliff community | Vegetation dominated by shrubs and ferns, on steep aspect, from lowland to montane elevations, in wet settings. Typical species include *Metrosideros, Coprosma, Vaccinium, Machaerina,* and *Dicranopteris,* Often adjacent to ‘Ōhi‘a Forest (uluhe) and ‘Ōhi‘a Forest (native shrubs) q.v. |
| Coastal strand vegetation | Open-closed canopy forest, most often at low elevations, part of a complex mosaic of naturalized alien vegetation in a mosaic of forest, shrubland and grassland. Small occurrences of native forest also may be found in this class. |
| Cultivated agriculture | Planted lands of variable physiognomy, with annual to multi-year stability. May include ordered rows of tree plantings (often dense) in agricultural-zoned lands, typically in lowland mesic and wet settings. May include a wide variety of dominants, including macadamia, coconut, banana, guava, papaya, sugarcane, pineapple, coffee, vegetable crops, and fallow fields. |
| Developed | Adopted with minor changes from the Coastal Change Analysis Program’s Developed land cover class, thus defined: “Contains little or no vegetation. This subclass includes heavily built-up urban centers as well as large constructed surfaces in suburban and rural areas. Large buildings (such as multiple family housing, hangars, and large barns), interstate highways, and runways typically fall into this subclass." |
| Grassland | Uncharacterized mixed alien grasslands, often at low elevations, often as part of complex vegetation mosaics of grass, shrubs, and trees. This class includes large areas dominated by fountain grass (*Pennisetum setaceum*), buffel grass (*Cenchrus ciliaris*), kikuyu grass (*Pennisetum clandestinum*) and other species. This class also includes planted grass of parks and golf courses. |
| Kiawe dry forest and shrubland | Vegetation dominated by an open to closed canopy of the alien shrub/tree kiawe (*Prosopis pallida*), typically in dry coastal settings, with dense stands adjacent to fresh groundwater sources near sea level. Often with an understory of alien grasses (most often *Cenchrus ciliaris*), and grading into Koa haole Shrubland and/or Fountain Grass / Buffel Grass Grassland, |
| Mixed native-alien forest | Vegetation dominated by a mixture of alien and native trees, typically along the transition between native-dominated forest at higher elevations and Alien Forest below (q.v.) Heterogeneous signatures, often complicated by steep and complex terrain. |
| Native forest | Vegetation dominated by a closed canopy of native trees with varying understories dominated by hāpu‘u, native shrubs, strawberry guava or uluhe, generally on moderate mesic to wet slopes from lowland to montane elevations. Often found adjacent to and grading into Closed ‘Ōhi‘a Forest, Open Koa-‘Ōhi‘a Forest, and Open ‘Ōhi‘a Forest. |
| Shrubland | Highly variable mixed shrubland dominated locally by one or more species, including *Lantana camara*, koa haole (*Leucaena leucocephala*), christmas berry (*Schinus terebinthifolius)*, klu (*Acacia farnesiana*), and others. Often grading into agricultural plantings or mixed alien grasslands |
| Uluhe ferns and native shrubs | Vegetation dominated by a shrubland (technically a fernland) of uluhe (*Dicranopteris linearis* and/or other native mat ferns; e.g., *Sticherus*, *Diplopterygium*), generally on moderate and steep mesic to wet slopes from lowland to montane elevations, typically on windward island slopes. Often with varying presence of native shrubs and sparse native trees, typically including ‘ōhi‘a (Metrosdieros polymorpha). Often found adjacent to and grading gradually with Open ‘Ōhi‘a Forest (uluhe) q.v. |
| Water & wetland vegetation | Marshes and other wetlands dominated by alien obligatory wetland grasses or grass-like vegetation, shrubs, or trees, e.g., oriental mangrove (*Bruguiera gymnorrhiza)* and American mangrove (*Rhizophora mangle*). These areas have generally been heavily degraded by feral pigs and subsequently, original native vegetation has been almost completely replaced by alien plants. This class is primarily derived from the National Hydrography Dataset. |

**Table S6.** Mean (± SD) permeability values given to the three studied species in each of the habitats, as well as the mean weighting factor used to include the mosquito effect.

| **Habitat class** | **‘Amakihi** | **‘Apapane** | **‘I‘iwi** |
| --- | --- | --- | --- |
| Alien forest | 0.67 ±0.19 | 0.58 ± 0.24 | 0.37 ± 0.29 |
| Coastal strand vegetation | 0.37 ± 0.37 | 0.25 ± 0.29 | 0.12 ± 0.24 |
| Cultivated agriculture | 0.10 ±0.11 | 0.07 ± 0.10 | 0.03 ± 0.05 |
| Developed | 0.16 ± 0.16 | 0.13 ± 0.18 | 0.01 ± 0.04 |
| Grassland | 0.23 ± 0.36 | 0.14 ± 0.21 | 0.09 ± 0.18 |
| Kiawe dry forest and shrubland | 0.33 ±031 | 0.23 ± 0.31 | 0.14 ± 0.27 |
| Mixed native-alien forest | 0.93 ±0.10 | 0.86 ± 0.17 | 0.66 ± 0.29 |
| Native forest | 1.00 ±0.00 | 1.00 ± 0.00 | 1.00 ± 0.00 |
| Cliff community | 0.68 ±0.28 | 0.72 ± 0.31 | 0.52 ± 0.37 |
| Shrubland | 0.67 ± 0.33 | 0.57 ± 0.31 | 0.34 ± 0.37 |
| Uluhe ferns and native shrubs | 0.77 ±0.20 | 0.62 ± 0.35 | 0.44 ± 0.41 |
| Water & wetland vegetation | 0.04 ±0.05 | 0.03 ± 0.05 | 0.00 ± 0.00 |
|  |  |  |  |
| **Weighting factor** |  |  |  |
| No mosquito | 1.00 ± 0.00 | 1.00 ± 0.00 | 1.00 ± 0.00 |
| Border (mosquito present in hot season) | 0.77 ± 0.17 | 0.63 ± 0.19 | 0.25 ± 0.31 |
| Always mosquito | 0.52 ± 0.26 | 0.37 ± 0.25 | 0.08 ± 0.25 |

**Table S7.** Summary of song characteristics by study area and species. We show the number of selections made, total number of syllables, the range in the number of syllables in the different study areas, percentage of exclusive syllables (i.e., syllables that only appear in one area) and average dissimilarity (i.e., average value of all pairwise dissimilarities in repertoire among study areas) for each species and study area. Pww: Pu’u wa’a wa’a

| ‘Amakihi | Hakalau | Kipuka | Kohala | Volcano | Pww |
| --- | --- | --- | --- | --- | --- |
| **Number of selections** | 187 | 388 | 154 | 131 | 313 |
| **Number of syllables** | 14 | 12 | 20 | 18 | 19 |
| **Range** | 2-6 | 2-6 | 3-8 | 2-5 | 2-9 |
| **Num. exclusive syllables** | 3 | 4 | 9 | 10 | 6 |

| ‘Apapane |  |  |  |  |  |
| --- | --- | --- | --- | --- | --- |
| **Number of selections** | 1128 | 1844 | 940 | 1688 | 1047 |
| **Number of syllables** | 71 | 70 | 70 | 105 | 83 |
| **Range** | 3-36 | 13-34 | 21-42 | 24-56 | 12-47 |
| **Num. exclusive syllables** | 10 | 22 | 18 | 38 | 30 |

| ‘I’iwi |  |  |  |  |  |
| --- | --- | --- | --- | --- | --- |
| **Number of selections** | 1355 | 376 | 381 | 50* | 904 |
| **Number of syllables** | 94 | 51 | 41 | - | 59 |
| **Range** | 20-47 | 2-28 | 7-20 | - | 7-36 |
| **Num. exclusive syllables** | 27 | 5 | 5 | - | 15 |

*Because of the low sample size for ‘I’iwi at Volcano, we did not include this site in the analyses.

Table S8. GLMs relating the Sørensen dissimilarity with the maximum and minimum resistance values.

| **Species** | Value | **Coefficient** | **p-value** | **R^2^** |
| --- | --- | --- | --- | --- |
| **‘Amakihi** | Maximum | -0.368 | 0.663 | 0.025 |
|  | Minumum | -0.197 | 0.529 | 0.051 |
| **‘Apapane** | Maximum | -0.011 | 0.960 | 0.001 |
|  | Minumum | -0.011 | 0.880 | 0.003 |
| **‘I‘iwi** | Maximum | 0.042 | **0.039** | 0.696 |
|  | Minumum | 0.009 | **0.019** | 0.783 |

**Figure S1.** Connectivity degree of the Hawaii Island for the ‘Amakihi (left column, a,d,g), ‘Apapane (middle column, b,e,h) and ‘I’iwi (right column c,f,i) using the mean of the permeability values estimated by the experts (first row, a-c), the mean + SE (second row, d-f) and the mean – SE (third row, g-h). The red dots are the location of the recorders at the five study sites.


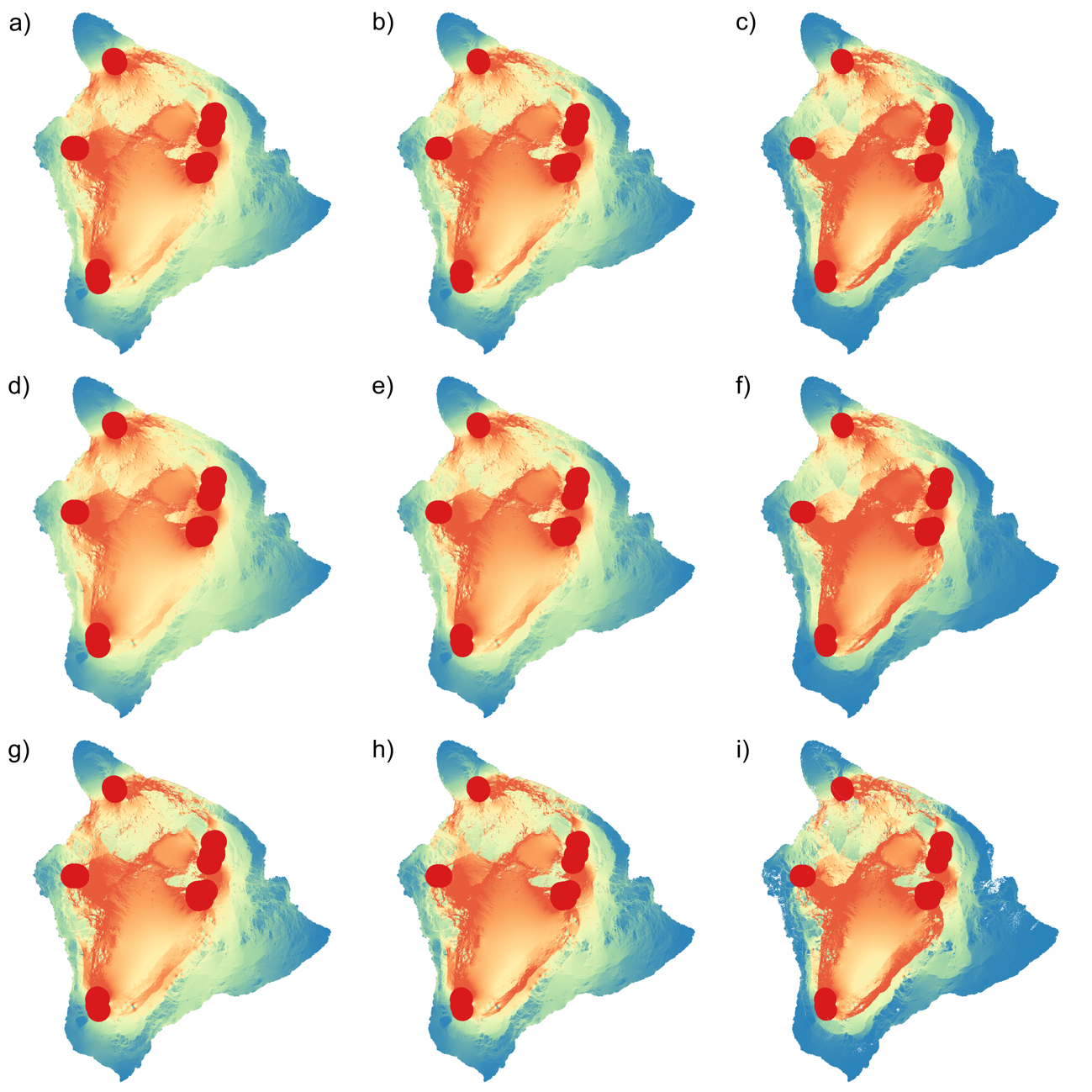

Supplement: Supplementary file 1 — Data S1: ece371919‐sup‐0001‐Supinfo.docx. [file ECE3-15-e71919-s001.docx]
